# Patient Care via Video Consultations: Piloting and S.W.O.T. Analysis of a Family Medicine Digitally Synchronous Seminar for Medical Students

**DOI:** 10.3390/ijerph19158922

**Published:** 2022-07-22

**Authors:** Franziska Särchen, Susanne Springborn, Achim Mortsiefer, Jan Ehlers

**Affiliations:** 1 Faculty of Health, Witten/Herdecke University, 58455 Witten, Germany; 2 Allgemeinmedizin Breckenheim, 65207 Wiesbaden, Germany; springborn-komissarenko@t-online.de; 3 Chair of General Practice II and Patient-Centeredness in Primary Care, Faculty of Health, Witten/Herdecke University, 58455 Witten, Germany; achim.mortsiefer@uni-wh.de; 4 Didactics and Education Research in the Health Sector, Faculty of Health, Witten/Herdecke University, 58455 Witten, Germany; jan.ehlers@uni-wh.de

**Keywords:** telemedicine, digital health, video consultation, general practice, family medicine, education, medical students, distance learning

## Abstract

Background: There is a need to familiarize medical students with the specifics of video consultations. This paper presents the concept and tests of a digitally synchronous distance seminar in which medical students practice video consultations as an aid to a family physician’s activity in a patient-oriented manner. The aim of the evaluation was to analyze the strengths, weaknesses, opportunities, and threats (S.W.O.T.) of the teaching concept. Methods: A total of 12 students carried out video consultations independently and under medical supervision. The seminar included two elements: (A) All students and teachers were video consulted in a family practice; (B) A small group performed a video consultation in a patient´s home environment. The students’ evaluation was conducted with two questionnaires (pre/post), which were analyzed with descriptive statistics and qualitative content analysis. The S.W.O.T. analysis was elaborated by the author team based on the results of the questionnaires and the interviews with the teachers. Results: Students learned the limits and possibilities of teleconsultations and deepened their family medical knowledge. Strengths: Among others; increase interest in video consultations, patient contact, focused work. Weaknesses: Among others; technical difficulties and the time it requires. Opportunities: Among others; involve students with multiple workloads in patient teaching. Risks: Among others; no integration into the curriculum yet, few personnel resources. Conclusions: The learning model familiarizes medical students with competences in family medical patient care using video communication. The results of S.W.O.T. analyses can be weighted differently. Project groups can decide individually if they want to integrate the learning concept into their curriculum and which further improvements are necessary.

## 1. Introduction

Video consultations are an aid in family medical patient care. This allows patients to communicate online in real time with their primary care physician while in their home environment. Eliminating the need to travel to an office can save patients time, money, and stress, as well as improve the access to care in rural areas for patients with mobility limitations and/or long travel distances to the office [1,2]. In addition, the physical distance protects against the transmission of infections [3]. Family physicians are increasingly offering video consultations to their patients. In particular, the number of consultations has increased since the onset of the COVID-19 pandemic [4,5,6,7,8]. 

Video consultations can also be performed with the help of non-physician health professionals. Thus, patients who do not have technical tools, who wish to have contact persons on site, or who need an examination beyond self-diagnosis, can be included. The specialist examines the patient in the practice or at home, while a physician is connected via video consultation [9].

In both forms of telemedical patient care, the physician faces similar challenges. This is because the type of interaction between physicians and patients via video differs from analog face-to-face consultations. For example, in video consultations, there is detailed communication about self-diagnosis and management of technical problems [10]. In contrast to face-to-face consultations, physicians must describe the activities they perform outside of the patient’s field of view [10].

Future physicians can familiarize themselves with the implementation and specifics of video consultations already during their studies. Different teaching methods are offered for this purpose. For example, medical students can learn how to perform physical examinations remotely on standardized patients [11]. The establishment of a trusting relationship between patients and students within video consultations is also taught [12]. Interaction with real patients from a distance is made possible for medical students with, for example, the help of cameras or robots that accompany experienced physicians during hospital rounds [13,14,15]. Medical students also accompany patient cases in psychiatry, cardiology, internal medicine, pediatrics, and neurology via video consultation [16,17,18]. There is no consistent curriculum in family medicine to learn telemedical patient care. There are only a few known learning interventions [19]. For example, Bhatia et al. [20] describe a face-to-face clerkship that was replaced by video consultations during the COVID-19 pandemic. Both patients and students were in their home environment [20]. The students only experienced the medical activity in the patients’ home environment. In addition, the students were mainly responsible for preparing the video consultations and, in the last phase only, for conducting the consultations. The students did not evaluate the project.

To the best of our knowledge, no learning method is known in which medical students learn family medical video consultations within a digitally synchronized distance learning seminar, conducting patient consultations in the patients’ home environments and within a practice from the very beginning. We conducted such a learning project. The additional learning environment of the family medicine practice was to show the students the diagnostic possibilities. We opted for a digitally synchronous remote seminar so that all participants would have flexibility in terms of location and would be protected from infection. The seminar should be synchronous to ensure lively discussions and real patient contact. This paper presents the design and trial of such a learning concept. In addition, student evaluations were obtained at the three levels of family medicine, video consultations, and didactics. A S.W.O.T. analysis (strengths, weaknesses, opportunities, and threats) was also conducted to assess the learning concept.

## 2. Materials and Methods

### 2.1. Study Design and Research Objectives

The teaching project consists of two elements: (A) A group of students and teachers is video-consulted into a family practice with a patient; and (B) A small group performed a video consultation with connection to the home environment of a patient. The experiences from these two elements will be subsequently described and compared. For this purpose, a structured processing of protocol data of the seminar hours and experience reports of the participating students was carried out. 

In addition, the following research questions were pursued on three levels:

- Telemedicine research level:
  1. To what extent can telemedicine skills be fostered in students participating in the seminar?
- Family medicine research level:
  2. To what extent can family medicine competencies be promoted among students participating in the seminar?
- Didactic research level:
  3. How is the didactic concept of the teaching project assessed by the participating students?
  4. What are the strengths and weaknesses of the learning concept according to the participating students and teachers?
  5. What are the opportunities and risks for the future?

### 2.2. Participants of the Seminar

The voluntary pilot project was designed for 12 medical students (7 women, 5 men) of the University of Witten/Herdecke (Witten, Germany) in June 2021. The students were recruited via an email distribution list from semesters 6 to 11. A family physician from a teaching practice at the University of Witten/Herdecke, who offers video consultation hours to her patients, was involved in the pilot project. A total of 12 patients were suggested by her. The patients suffered from different chronic diseases, e.g., diabetes, depression, and heart failure. The patients were also selected on the basis of the regular consultation appointments with the family physician taking place for half of the patients via video consultation.

Written informed consent was obtained from both students and patients after the educational interviews for participation in the pilot project. The primary care physician led the telemedicine content of the seminar and took responsibility for the patients. An academic and specialist in family medicine took over the management of the seminar sessions and encouraged the students to think in terms of family medicine. There was also a project leader and a doctoral student as organizer and recorder, respectively.

Students and teachers each had to have terminal equipment with internet connection, as well as video and audio transmission.

The experimental procedure was approved by the ethical committee of the University Witten/Herdecke (S-54/2021).

### 2.3. Teaching Project Concept

Teaching format, learning objectives, and concept, as well as the possible incorporation into the university curriculum, were discussed within a team of students and experts responsible for education at the University of Witten/Herdecke.

**Figure 1:**
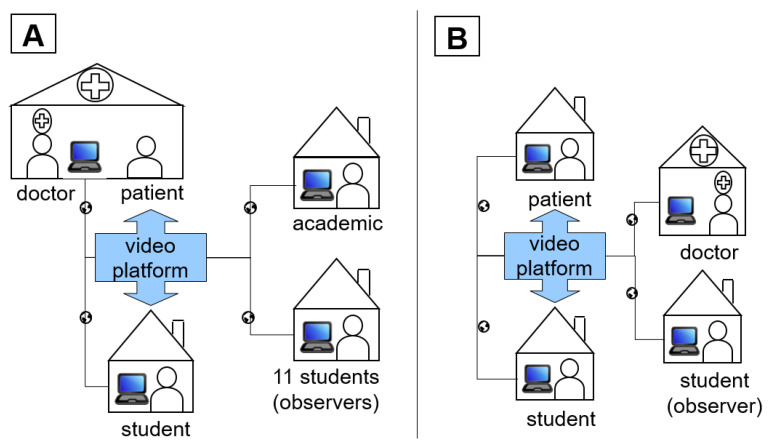
Spatial and personnel structure of group appointments/element A; (**A**) individual appointments/element B (**B**).

The pilot project was planned for a period of 4 weeks in June 2021. All students had 5 appointments. The first appointment was an introductory session via Zoom (Zoom Video Communications Inc., Denver, CO, USA). Within the 3 group appointments, that followed at intervals of one week, half of the patients were invited to the practice (Figure 1A). The other half of the patients and all the students were each given the access link to one of 6 individual appointments in which the patients were in their home environment (Figure 1B). Each student had to perform a consultation under the supervision of the physician. For this purpose, the meeting room platform comjoodoc (comjoo business solutions GmbH, Berlin, Germany) was used within the group appointments (element A). Within the individual consultation (element B), the video consultation service arztkonsultation.de (arztkonsultation ak GmbH, Schwerin, Germany) certified by the health insurer was used.

The compulsory dates for the introductory meeting and the 3 group appointments were set by the teachers before the start of the seminar and communicated to the students. The patients were asked the group appointment date on which they preferred a consultation. Two consultations took place per group appointment. For the individual appointments, the doctor set up 6 appointed times. For the coordination, patients and students were independently asked early on which of these 6 appointments they could attend in order to fill each slot with one patient and two students.

Students had the opportunity to prepare for their interview through pseudonymous patient records. Students received a certificate of achievement for attending 5 seminar sessions.

The introductory meeting included getting to know each other, presentation of the learning objectives, a tour of the practice and a discussion about the organization. One goal was to motivate the students to participate in the pilot project. Technically, a short lecture defined the term video consultation, mentioned its current use and advantages, the legal and technical requirements, and the costs. Via the video camera, the family physician demonstrated to the students with which facial expressions certain emotions are evoked. The categorization of symptoms into different specialties was addressed.

**Figure 2:**
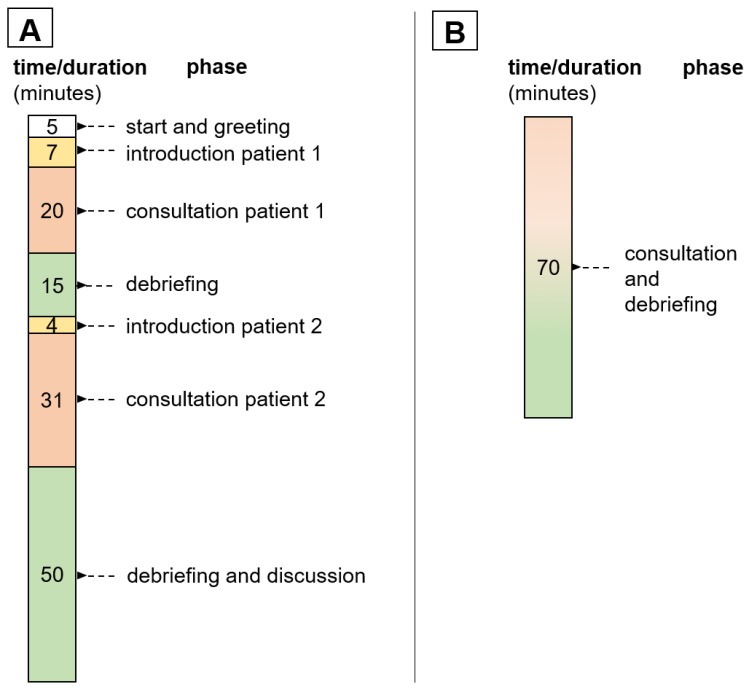
Timing of group appointments/element A; (**A**) individual appointments/element B (**B**).

In the 3 subsequent group sessions (element A), the focus was on performing, pre-discussing, and post-discussing two patient cases each. In this process, the entire group was video called into the primary care physician’s office at weekly intervals. The schedule of the group appointments with the temporal mean values is shown in Figure 2A.

The introduction of the patient was conducted by the student who performed the video consultation. For this purpose, the person shared their screen and thus showed a self-created presentation with the most important data from the patient’s file. After the questions of the lecturer and of the other students were clarified, the physician was notified. The video transmission from the doctor’s office was started and showed the patient’s face. The student performed a consultation with the patient. In the subsequent debriefing, the academic asked for the student’s self-assessment of the consultation, and a professional discussion of the illnesses took place. A second patient presentation, consultation, and debriefing followed. This was conducted by another student member of the group. Finally, there was a discussion of diagnostic findings, the students’ observations of the specifics of telemedicine communication and perception, and didactic suggestions for improvement. The physician joined this discussion as soon as the patient left the practice.

Within the individual consultations (element B), in contrast to the group appointments (element A), only one patient consultation took place. The physician supplemented these consultations with a debriefing session. The average duration of these seminar appointments is shown in Figure 2B.

A more detailed account of the organizational steps and templates for documents to follow up the seminar are included in additional file S1.

### 2.4. Evaluation and Protocol

The participating medical students were interviewed and observed at three measurement time points. Part 1: Prior to the start of the seminar, an online questionnaire was used to assess the students’ attitudes toward the upcoming teaching method and the content, including learning objectives (response selection procedure, scale responses, free text; see additional file S2). Part 2: At each seminar date, students were able to summarize what they had learned on a digital white board (MIRO (RealtimeBoard Inc., San Francisco, CA, USA) or provide feedback orally during group meetings by phone, or by e-mail. Part 3: The overall evaluation was collected after completion of the four-week seminar through a second online questionnaire (response choice method, scale responses, free text; see additional file S3). The aim was I. to get the evaluation about the structure and the satisfaction to the completed seminar, II. to uncover effects on the students personally, and III. to uncover the attitude to the future design of such a seminar.

The questionnaires were created using the web application soSci Survey (SoSci Survey GmbH, Munich, Germany) and contained a total of 42 items. Of these, 14 were open-ended questions. For 8 items, students could rate them using a slider. This corresponded to a Likert scale, the endpoints of which were the categories “strongly disagree” (1) and “strongly agree” (101). Prior knowledge and interest in the topics of the seminar were asked of the participating students by means of a Likert scale before the start of the seminar. The rating scale contained 5 scale points. The final points were labeled with the categories “does not apply at all” (1) and “applies completely” (5).

The schedule of group appointments and the total duration of individual appointments were recorded.

Data from the online questionnaire, digital white board, and feedback in the seminar from emails and phone calls were collected for qualitative descriptive analysis in tabular format. Key messages were extracted by categorizing them into headings.

### 2.5. S.W.O.T. Analysis

Subsequent to the seminar, the team of authors worked out the strengths, weaknesses, opportunities, and risks of the teaching method. For this purpose, the internal view of the participating teachers was determined through interviews after the pilot project was completed. The external view of the participating students was collected by evaluating the online surveys.

## 3. Results

All the students (*n* = 12) completed the questionnaires.

### 3.1. Telemedicine Research Level

**Figure 3:**
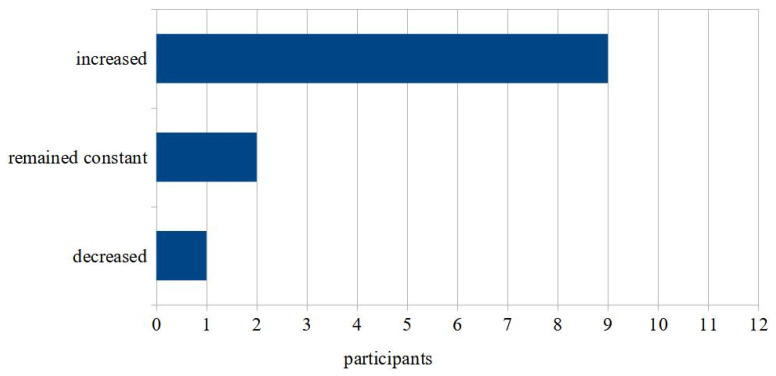
Evaluation of the statements within a single choice for the following statement: “My interest in remote treatments is due to patient discussions…”.

Interest in video consultations increased for many as a result of the patient interviews (see Figure 3). The participating students’ interest in video consultations was predominantly high before the seminar began (5-point scale: median: 4, max: 5, min: 2). Most students denied having much prior knowledge on the topic (5-point scale: median: 2.5, max: 4, min: 1). Of the 12 participants, 11 agreed that the distance learning seminar complemented their previous teaching on telemedicine (median: 101, max: 101, min: 98 + one outlier: 3). 

The participants described their learning successes in the field of remote treatment in detail and with examples. They named requirements for remote treatment including technical equipment. They stated that they had experienced its limits and possibilities. They compared their experiences within video consultations with their previous experiences from face-to-face consultations. Video consultations were described as a “good complement to face-to-face consultations” (comment of one participant). Two participants stated that they found it easier to focus on the structure of the conversation than they did in face-to-face consultations.

The students elaborated on the communicative specifics within video consultations. For example, it was important for physicians to speak slowly and maintain eye contact. At the beginning of a consultation, the student who conducted the consultation introduced him/herself to the patient and asked about the sound and image quality. The students circumvented communication problems during technical difficulties with the sound transmission by summarizing and repeating the content of the patient’s conversation. 

They learned to assess the limits and dangers of video consultations and the associated perceptual deficits, and to involve the patient in a face-to-face consultation or emergency medical care for appropriate medical concerns. 

Students had the opportunity to compare the video consultations of the group appointments within the practice (element A) with the video consultations connected to the patient’s home environment (element B). One student noted that different family medical conditions require different care, and the office is not always the best place for this. On the other hand, video consultations within the family medicine office (element A) also offered advantages. In this setting, selected students were able to actively participate in the consultation, take patient histories, and learn about the use of digital diagnostic tools remotely via instructions to the attending family physician. For example, the students asked the doctor to perform a digital 1-lead ECG on a patient. After a few seconds, the entire group was able to view the findings online. Other diagnostic equipment in the practice was also used, e.g., sonography device, tuning fork, blood pressure monitor, and scales, which were presented to the students via the front camera of a tablet during the reporting of findings. In 4 out of 6 consultations, students asked the doctor to hold the camera closer to specific skin sites.

Within the final discussion at the end of the group appointments (element A), students compared their perception of the situation with the perception of the physician on site. For example, students would not have perceived the signs of shortness of breath via video transmission. Other topics at the end of the group appointments included nonverbal means of communication during video consultations, dealing with preventable dangerous courses, and reporting impressions within individual appointments (element B).

As a suggestion for improvement, students mentioned that a demonstration of a video consultation by an experienced physician during the introductory session would be useful.

### 3.2. Family Medicine Research Level

**Figure 4:**
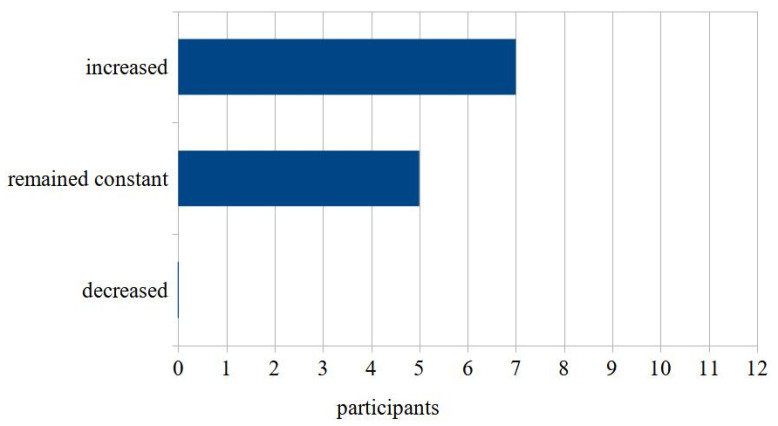
Evaluation of the statements within a single choice for the following statement: “My interest in family medicine is due to the patient interviews…”.

The interest in family medicine increased in 7 of the 12 participants as a result of the patient interviews (Figure 4). Before the start of the seminar, the students indicated a high interest in family medicine (5-point scale: median: 4, max: 5, min: 3). Three participants stated that they wanted to become a specialist in family medicine. Most participants could neither agree nor disagree with the statement that they had a lot of prior knowledge about family medicine (5-point scale: median: 3, max: 4, min: 2). For the statement that the distance learning seminar complements the participants’ previous teaching about family medicine, agreement was heterogeneously distributed on the Likert scale (median: 77, range: 94).

When asked about what they learned, students indicated that they deepened their family medical skills, knowledge, and working techniques. They gained patient-oriented insights into various family medical clinical pictures. The patients came to the teaching consultation for routine examinations/interviews, but acute atrial fibrillation was also diagnosed. The students indicated, as a suggestion for improvement of the teaching project, to include more acute treatment occasions in the seminar.

The students deepened their knowledge of conducting a conversation and taking medical history. They also learned how to view patients holistically. Some students reported back after the individual appointments (element B) that the patients were open to them. Some students succeeded in establishing a stable relationship of trust with the patients.

Within the professional debriefing of the group appointments (element A), among other things, further diagnostics of disease patterns of the patient cases were addressed. The pathological background and complications of the corresponding clinical pictures were also integrated into the debriefing. In the subsequent discussion of the group appointments (element A), for example, anamnesis questions for initial interviews were discussed. One student wished for an even more detailed discussion of patient cases.

As another learning outcome, one student stated that he had learned how to handle complex patient records to obtain an overall picture of a person through the seminar.

### 3.3. Didactic Research Level and S.W.O.T. Analysis

All participating students agreed with the statement that attending the event had been worthwhile for them (median: 100.5, max: 101, min: 78). The distance seminar was an appealing method of digital teaching (median: 95.5, max: 101, min: 76).

**Figure 5:**
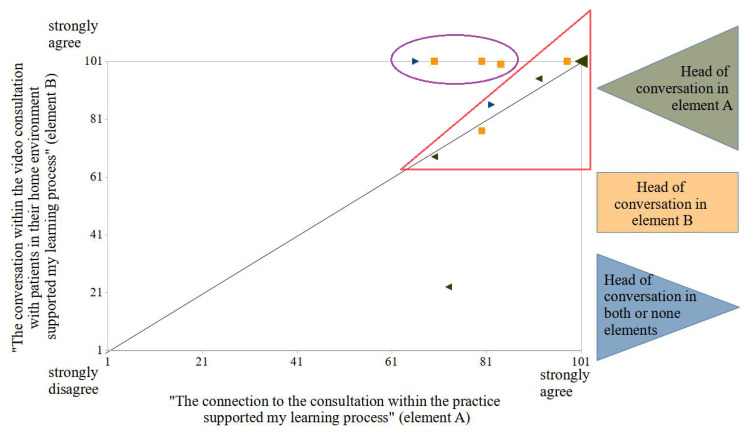
Correlation of two 101-point Likert scales to the following statements: “The connection to the consultation within the practice supported my learning process” and “The conversation within the video consultation with patients in their home environment supported my learning process”.

The students particularly praised the opportunity for patient contact. All students found the consultations supportive of their learning process. The participants’ agreement was asked regarding the following two statements: “The connection to the consultation within the practice supported my learning process” (element A, group appointment) and “The conversation within the video consultation with patients in their home environment supported my learning process” (element B, individual appointment). The correlation of the results of these two items is shown in Figure 5.

The statements from 7 of the 12 participants show a strong positive correlation (see graphics within the red triangle in Figure 5). Three of four students who agreed significantly more with the second statement had their discussion lead to a patient consultation during a one-on-one appointment (element B, indicated in yellow within the purple circle in Figure 5).

Five of the six one-on-one planned appointments took place. In each case, one student led a consultation with an adjunct in the patient’s home environment, while another student and the primary care physician observed. Some of the students reported that they had felt comfortable, especially in the small group of four persons.

**Figure 6:**
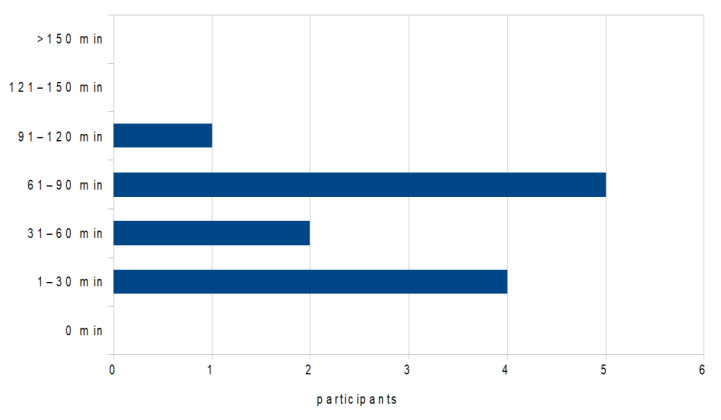
Evaluation of the data within a single choice to the following question: “How much time did you invest in the preparation and follow-up of patient consultations in which you were asked to lead the conversation?”.

The workload for the students has been rated by all as “just right”. The compulsory attendance for the students during the total of five seminar dates was approx. 11 h. The preparation time based on the files as well as the time for post-processing of the seminar dates could be chosen individually by the students. The time for preparation and post-processing of the appointments without leading the discussions was given by the students as a maximum of 30 min. The estimation of the working time for the appointment with discussion leadership is shown in Figure 6.

The students cited the length of the group appointments as a point of criticism and mentioned suggestions for improvement such as expanding the number of seminar hours or halving the number of consultations per appointment. 

The working atmosphere was rated as “good” by 10 of the 12 participating students (median: 99, max: 101, min: 77 + 2 outliners: 8, 54). The other two participants mainly criticized a lack of appreciation of the doctor’s treatment methods, the high number of lecturers, and a high noise level.

Some students criticized the technical difficulties. Differences in picture and sound quality between the participants, problems sharing the screen, and time delays were observed. One student expressed the wish for better technical means to establish more contact with the patient while the doctor is performing the examination. The group developed some solutions to the technical difficulties during the seminar (Table 1). In addition, the team is in contact with the technical support of the software programs and is looking for alternatives for digital-synchronous diagnostics.

Future student participants should be motivated, technically equipped, and have prior experience in family medicine, according to the students (see Table 2).

In the following section, the key points of the S.W.O.T. analysis from Table 3 are explained and questions 4 and 5 are answered.

#### 3.3.1. Strengths

The teachers see the strengths of the learning method in the patient contact which complements the classroom teaching. The students learn practically and authentically at the places where family medicine is practiced by means of video transmission: in the practice (element A) and in the home environment (element B). Without travel, this teaching format offers students the opportunity to conduct professional patient interviews and examinations. The new German licensing regulations call for both the strengthening of family medicine and the strengthening of digital medicine [21]. The teaching format offers an opportunity to familiarize medical students with the family medical communication technology of video consultation hours and thus meets the requirements. 

The participants in the pilot project would have captured the concerns of patients who were in a different region of Germany and whom they had previously only known from files. The results of the evaluation showed the satisfaction of the students with the pilot project and the learning success. The pilot project shows that communication and relationship-building with patients is possible digitally, even on intimate topics. From the point of view of the lecturers, students can be introduced to telemedicine with this teaching method. The strengths and limitations of video consultations were demonstrated and expanded. The family physician had also improved her approach within video consultations.

The students especially praised the commitment of participants and lecturers. The lecturers see the staffing and the appreciative interaction with each other as a strength of the pilot project. This included the participation of committed, empathetic, patient patients who were willing to talk to a student in front of many viewers, some of whom had previous experience with student teaching via video communication. The students were willing to communicate, committed, and very well prepared for the patient consultations, even though students who were primarily interested in obtaining a certificate also participated in the pilot project. This suggests a high intrinsic motivation for the field of interest of the voluntary course. They had been willing to invest time beyond the mandatory courses and demand a more time-intensive expansion of the teaching model. The students’ commitment was evident in the number of suggestions for improvement they noted. Through these, the teaching method could be improved together with patients and teachers. For example, the optimal position of the camera, lighting, and patient chair within the consulting room could be worked out. Also, new technical devices (e.g., for digital vital parameter measurement) could be integrated into the seminar. The strength within the supervision of the students was due to the commitment with which the lecturers carried out the pilot project. This included the openness to change the concept as well as the implementation of a feedback culture. The small group size (2–12 students) had also intensified the supervision. Particularly in the individual appointments (element B, two students), questions were addressed individually and discussion techniques were practiced.

The family physician’s conviction that the care of patients can be sustainably improved by means of video consultations had shaped the course of the seminar. The knowledge resources of the lecturers include several years of experience in family medical practice, the implementation of video consultation hours, and the implementation of university teaching. Another empowering resource was the amount of time the faculty invested in organizing and the amount of time the students and patients invested in implementing the pilot project. The digital format of the course allowed for focused work, especially due to the reduction of language and the limited image section of the participants, as well as the reduced sensory impressions from their own home environment.

From the external perspective of the students, the patient contact from the position in charge of the interview as well as the observer role was praised above all. In particular, the responsibility and freedom granted to the students with regard to the design of their patient interview and the preparation and follow-up were positive.

The students state that they have learned the difference between face-to-face contact with patients and contact via video consultation. Working remotely made it easier to focus on the relevant content of the course more than in a face-to-face setting.

The students see the small group size as an added strength. Especially in the individual consultations (element B), the atmosphere between the doctor, two students, and the patient was very pleasant and relaxed.

The results of the evaluation show that the students are satisfied with the event overall and that the teaching method can increase the interest of participating students for video consultations. The learning process in the team was seen as particularly positive by the students, as was the incorporation of suggestions for improvement. The combination of practice and theory is appealing. The preparation for the seminar including the provision of the files in advance was helpful.

When asked about the strengths of the project, the commitment and motivation of the teachers and students was stated within the evaluation.

The increasing relevance of video consultation was mentioned as a reason for participating in the seminar. The students see the trend that the digitalization of medicine is increasing and see the teaching format as an opportunity to deal with this and, in particular, to recognize its limits and opportunities.

#### 3.3.2. Weaknesses

Due to the lack of experience on the implementation of this teaching method, there were uncertainties among the teachers. In particular, the dimensions of the topics that would be of importance to students in this seminar could not have been estimated despite prior inquiry.

The instructors invested time in meetings about schedule coordination, testing of technology, and discussions of students’ comments. Additional organizational effort was required for the acquisition and education of patients and students. From the university side, time and costs are incurred for the activation of student access to the data-protected video platform. Additional costs are incurred for equipping the family physician’s practice with cameras, a tablet, a computer, a smartphone, a digital stethoscope, and access to a video platform certified by the health insurance fund. There would be an additional time commitment due to the individual setup of the platforms, e.g., uploading patient records and sending access links for students.

The teaching model is dependent on functioning technology. The technical problems that disrupted the course of the seminar were mentioned by the teachers as a weakness. These were partly compensated by improvements. Nevertheless, it could not be guaranteed that, for example, all participants in the seminar would be able to speak to the patients without delay, that examination findings would be transmitted, and that, for example, findings of the skin could be viewed in realistic image resolution. More intensive preparation and tests before the start of the teaching project could have prevented some of the problems that occurred. Depending on the demands, an acceptance of the current transmission possibilities is conceivable.

The teachers see the teaching model as a supplement to face-to-face teaching. Especially due to the greater perception by means of touching and smelling, the face-to-face contact with patients is indispensable for the students.

One lecturer criticized the lack of an emergency checklist for video consultations with the students. In addition, the time frame did not allow for a detailed discussion of treatment needs besides the exploration of the peculiarities of video consultations. In addition, some seminar appointments exceeded their agreed-upon duration. Nevertheless, a larger number of patient-side discussions would be desirable.

When patients are cared for by two specialists, there is a variance in the style of treatment and the course of treatment. This requires acceptance, clear communication about the areas of responsibility, and possible debriefing of professional differences.

Communicating remotely with participants could lead to a loss of information, according to faculty. A lack of accessibility by phone or unread emails makes remote communication difficult.

According to the teachers, there was no guideline for high-quality communication within teleconsultations, which could be used as an orientation for the lessons.

The students see the weaknesses of the pilot project primarily in the scope of learning, in the absence of time, and in the technical difficulties. They criticized the scope of learning within the group appointments for being too large and the family medical focus for being unclear when the appointments were too long. More frequent and shorter (<2.5 h) appointments could be a solution approach. However, the amount of time given by the lecturers cannot be reconciled with the number of patient interviews the students would like to conduct.

The students suggested including patients with acute treatment needs in the teaching project. Inviting patients on a long-term basis reduces the likelihood of acute treatment occasions.

The students wished to expand the telemedical learning environment with a view to the future and in comparison with other countries. The scope of learning on the topic of data protection was too small for the students. They also called for the improvement of technical equipment.

#### 3.3.3. Opportunities

The teaching method enables medical students and future experts from other health care professions to work with patients without the risk of infection. The teachers also consider the seminar to be useful outside of pandemics. Likewise, the participating students within the online survey agreed with the statement that the seminar should also be offered after the COVID-19 pandemic (median: 101, max: 101, min: 85).

The teaching method could lead to more inclusion. In particular, students with children, part-time jobs, and long travel distances may benefit from remote patient instruction from the students’ perspective. The core points of the teaching concept (patient contact, family medicine, digitalized medicine, local flexibility, and study in small groups with debriefing) can also be implemented in alternative formats, according to the teachers. One possibility is to conduct the patient discussions as before and to integrate them into a learning context, e.g., in the form of an internship following a lecture series or eLearning unit on chronic diseases, in order to practice what has been learned theoretically. The internship could be carried out as a digital family medicine internship, in which a student accompanies a family physician during (video) consultations. This is also possible as a classroom method or blended learning concept. Getting to know the participants in presence could simplify the cooperation.

The implementation of the video consultation could be distributed among several family physicians. The digital format means that universities are not limited to collaboration with teaching practices in the immediate vicinity and can expand their network. However, according to faculty, collaboration with familiar teaching practices could facilitate exchange on didactics and content.

Another idea for the modification of the previous seminar concept is the graduation in difficulty levels. Depending on their previous knowledge, the students could first practice anamnesis in video consultation, then practice digital examination methods, and in a further step they could be taught how to interpret long-term values from digital health data. It would be possible, for example, to integrate newly developed apps into the teaching concept.

The teaching concept offers the opportunity to apply the skills learned for conducting a video consultation in other disciplines as well. The seminar concept could also be applied in a modified form in other medical specialties to teach telemedical competencies to medical students.

As a team, students, teachers, and patients can further develop the concept and motivate other physicians, patients, and students to conduct video consultations. The joint use of video consultation hours and the exchange of suggestions for improvement as well as corresponding research could lead to a qualitatively better health care of the patients. For example, by improving the technical conditions and developing emergency checklists.

There are gaps in research, particularly with regard to the didactic format, the implementation of high-quality video consultation hours, and the sensible use of digital medicine in general. The use of digital medicine is less widespread in Germany than in some other countries. Video consultations would offer the chance to relieve the shortage of specialists in rural regions of Germany. Through the described teaching method, future physicians could decide whether they would like to use digital medicine or not.

#### 3.3.4. Risks

The teachers see the risks of the teaching method in the integration into the curriculum of the respective universities. This requires commitment, money, and time, which is not available in every project group. If the participating lecturers, students, or patients are not motivated, there is a risk that the seminar will not take place or that the learning success will be lower. It remains uncertain whether other patients, teaching practices, and students are willing to cooperate in the teaching project. Especially patients without experience with distance learning. Performing family medicine under the observation of several students and other specialists is an unusual way of working. Therefore, the willingness of teaching physicians to participate is questionable.

In the case of professional differences between two specialists participating together, team-building and the learning success of the students are endangered. Another risk is the focus on telemedicine instead of using it only as a tool for family medicine.

The use of technical aids in teaching could lead to communication problems.

The flexibility of the workplace presents the risk that people from the public sphere participate and thus data protection is not guaranteed.

## 4. Discussion

The concept of a digitally synchronous distance seminar in which medical students learn to perform video consultations close to the patient as an aid to a family physicians activity was tested within this innovative pilot project, according to the planning.

The first research question was to what extent can telemedicine competencies be promoted among students participating in the seminar. The competencies acquired include digital synchronous communication during family medical patient consultations. Students prepared, conducted, and rehashed successful video consultations. This can be concluded from the comparison of the protocol data and information on learning success with Jiménez-Rodríguez et al. [22] criteria for conducting a successful video consultation. The results of this study show that there is a need for the students to learn telemedicine and video consultations. This coincides with current and future needs: telemedicine has established itself in the field of video consultation according to a representative survey of the German medical profession [23]. In addition, the catalogue of learning objectives for medical universities in Germany (NKLM) provides for telemedical competencies [24]. These range from patient-oriented application of the possibilities of explaining and considering advantages and disadvantages, opportunities, and limitations as well as evaluation of ECGs [24]: VII.2-13.1.5, VIII.6-04.3, VII.4-01.1.4, VIII.2-06.3.10, VIII.3-03.1.3, VIII.6-04.3.3. These competencies could be acquired by the students participating in the seminar, according to the results of the study. Thus, the teaching method enabled the students to make an independent decision about whether and how they would like to offer this type of medical care to their future patients.

Recognizing the limits of telemedicine is one of the learning objectives of the learning objectives catalogue [24], VIII.6-04.3. This includes the technical difficulties we have identified in the weaknesses. Such problems are known in video consultations and there is a need to catch up [25]. The knowledge of the students regarding data protection could not be sufficiently promoted. This topic should be intensified in future teaching projects in order to protect future physicians and patients. In addition, our seminar is one of many telemedicine projects. These exist in a wide variety of disciplines and use a wide variety of methods from internships, lectures, simulation programs, app programming, etc. [11,13,14,15,16,17,18,19,20,26,27]. In the USA, “60 allopathic medical schools […] offer some form of telemedicine experience in their clerkship offerings” [27]. Our presented project complements this set of learning methods about telemedicine competencies in a patient-centered way but cannot teach all competencies.

The second question was to what extent could family medical skills be fostered in the students participating in the seminar. The medical students were able to practice communication with family medical patients. They stated that they had deepened their knowledge of how to take an anamnesis. The participants were also able to learn family medical work techniques and how to handle health records. Family medical skills such as performing a physical examination can be taught to a limited extent by means of distance learning. Compared to conventional face-to-face teaching, students cannot perform this activity themselves in the seminar. Instead, several students can observe the patient consultations including diagnostics. The heterogeneous evaluation of the statement that the seminar complements the previous teaching on family medicine is probably due to the diverse other family medicine teaching offers at the University of Witten/Herdecke [28]. The seminar should therefore not be used as a substitute for face-to-face teaching.

The topic complexes of family medicine and video consultations were planned to be equally time intensive; however, it became apparent during implementation that the students placed the focus of the debriefing on the topic complex of video consultations. This should be included in the planning of a next seminar.

The third question was how was the didactic concept of the teaching project assessed by the participating students. Overall, the students were satisfied with the course, the amount of work, and the working atmosphere. In contrast to the teaching method of Bathia et al. [20], the learning method described above consisted of two different elements of distance learning. Within the group appointments (element A), the entire group of students and instructors were video-consulted into a family medicine practice where a patient was present. Within the individual appointments (element B), a small group conducted a video consultation with a connection to the home environment of a patient. As an overall concept of both A and B elements, the pilot project led to an increase in interest as well as learning success with regard to family medicine and video consultations. The student feedback indicated that both A and B elements supported their learning similarly strongly. The group appointments (element A) allowed a greater number of students to experience the consultations and be involved in the discussions than in the individual appointments (element B). The diagnostic capabilities of the practice were also available for the group consultations. Participants stated that they liked the combination of the hands-on activity (led by the primary care physician) with the theoretical discussion (led by the academic). However, the size and duration of the group appointments (element A) had been too large while the time was too short compared to the individual appointments (element B) and thus the debriefing was more superficial.

According to the students, the learning environment within the individual appointments (element B) was more intimate and a stable relationship of trust could be established with the patients. According to the family physician, the questions could be answered more individually and the discussion techniques better practiced during the individual sessions (element B) than during the group sessions (element A). Due to the lack of co-teaching, the areas of responsibility were clearly defined. However, examinations beyond self-diagnosis, group discussions, and family medical teaching by the academic could not be conducted in this constellation. By integrating both A and B elements into the pilot project, students were able to compare the two sites of family medical patient care (practice and home environment) with advantages and disadvantages for the care of different medical conditions. It also allowed them to compare the learning environment. The results of the evaluation should thus be seen as feedback on the combination of element A and B. The wish of the participants to integrate the seminar into future teaching should be fulfilled. The evaluation of the students speaks for the fact that, in future projects, both the learning contents and both the A and B concepts should be maintained. However, a further development according to the students’ suggestions for improvement would be desirable, especially with regard to the timing of the group appointments and the technical implementation.

Overall, the S.W.O.T. analysis has shown that the assessment of students and lecturers is in agreement, especially regarding strengths and weaknesses. The analysis of the strengths, weaknesses, opportunities, and risks provides a basis for decision-making, according to which project groups can individually weigh up whether they should implement the learning model for themselves. If such a project were to be carried out again, the S.W.O.T. analysis would serve to optimize processes, eliminate weaknesses, and use strengths. Within the S.W.O.T. analysis, the teachers also pointed out alternative didactic concepts to convey the learning content. These could be tried out and explored in future projects. Compared to existing projects, the strength of patient contact (see S.W.O.T/ analysis) suggests that students prefer our concept to teaching through lectures (e.g., Agarwall [29]) or on acting patients (e.g., Cannon et al. [30] and others [11]). However, this needs further research. For other future projects, the results of the S.W.O.T. analysis can mean that independent, locally flexible team work on the patient can bring high satisfaction and learning success. With the weaknesses and risks mentioned, it is easier to plan what to look out for in future similar projects. The study results can serve as a basis for further research, both in terms of developing new teaching methods, for comparison with other teaching methods, and for quantitative surveys of students, teachers, and patients. The S.W.O.T. analysis also revealed research gaps, such as the lack of guidance on high quality communication within teleconsultations, which can be addressed in future research.

### Limitations

The pilot project was carried out on 12 medical students at the University of Witten/Herdecke and the lecturers were rated as very motivated by the students. This limits the transferability of the research results to other groups.

There is a lack of research on the attitude of the participating patients towards the teaching method. Such data were not collected due to resource constraints. Of the data that were collected, the results of the Likert scales of the questionnaires before and after the seminar cannot be compared. The reason is the different scaling (5- and 101-step). This does not affect the significance of the results presented in this paper.

## 5. Conclusions

The number of video consultations has increased during the COVID-19 pandemic. Video consultations will probably continue to be a means of medical care after the pandemic. Therefore, it is timely to integrate the competencies to perform this service into the education of future physicians. The corresponding teaching methods are diverse. Through the presented seminar, medical students will have another opportunity to deal with the future-relevant activity of digital medicine. The teaching method can complement the training to help them become contemporary family physicians. It presents many strengths and opportunities, weaknesses and risks. The future physicians acquire competencies in family medical patient care by means of video communication already during their studies. They will be able to assess the possibilities, but above all the limits, of video consultations when they offer them to their patients upon entering their professional lives. Therefore, in the opinion of the authors, the teaching model should be further expanded, disseminated, and scientifically investigated.

## Figures and Tables

**Table 1:** Technical problems and approached solutions.

Problem	Solution Approach
Picture blurred	Use of a tripod except for ultrasounds, otherwise the camera is too far away from the screen
Moderate picture quality	Optimized lighting (frontal/side)
View of patient from below	Tablet stands elevated on a tripod
Mouth–nose protection hides facial expressions and makes communication difficult	The primary care physician attends the meeting from another room during the history taking, so that the patients can remove their mouth–nose protection
Image section only on patient’s face	Second camera in the room, also to observe the gait pattern

**Table 2:** Analysis and categorization of free text answers to the following question: “What should future participants bring with them?”.

Category	Participants Answers
Prior knowledge	Medical history experiences Family medicine experience to help focus and recognize the difference between face-to-face and remote treatment Two completed family medicine internships Prior knowledge of internal medicine, especially pharmacological Broad general knowledge of various diseases from a variety of specialties
Setting	Empathy
	Motivation Appreciative attitude towards patients and teachers
Equipment	Stable internet connection
	Functioning terminal with good camera, microphone, headphones
	Quiet environment during patient interviews
	Second screen if necessary

**Table 3:** Strengths, weaknesses, opportunities, and threats (S.W.O.T.) analysis on the use of the described seminar in medical studies.

Strengths	Weaknesses	Opportunities	Threats
Patient contact (T, S)	Pilot project (T)	Lessons for times with & without pandemic (T, S)	Hurdles of incorporation into the curriculum (T)
Complement to face-to-face contact (T, S)	Costs (T)	Inclusion (T, S)	Lack of commitment (T)
Learning success (T)	Equipment (T)	Alternative teaching formats (T)	Lack of personnel (T)
Local flexibility (T)	Time required (T, S)	Integration into a learning context (T)	Professional differences (T)
Supraregionality (T)	Technical problems (T, S)	Incorporate teaching practices (T)	Technology problems (T)
Teamwork (T, S)	Limited perception (T, S)	Free choice of core topics (T)	Focus on telemedicine (T)
Adaptation of technology & learning objectives (T, S)	Extent of learning (T, S)	Application in other subject areas (T)	Data protection (T)
Human resources			
Preparation (T, S)	Lack of time (T, S)	Using & developing technological innovations (T)	
Voluntary event (T)	Limited treatment occasions (T, S)	Mutual motivation (T)	
Focused work (T, S)	Professional differences (T)	Improving patient care (T)	
Individualized design of one-on-one consultation hours (T, S)	Communication problems (T)	Closing research gaps (T)	
Satisfaction (T)	Data protection (S)	Support development of digital medicine in Germany (T)	
Relevance of video consulting hours (T, S)	Lack of quality guidelines (T)		

Legend: T = stated by teachers, S = stated by students.

## Data Availability

Questionnaire datasets generated and analyzed during this study are included in this published article and its supplementary information files. The steps for organizing such a seminar are included within the supplementary information files. Datasets generated during the current study are not publicly available due to the fact it contains identifiable sensitive data.

## Supplementary Materials

The following supporting information can be downloaded at: https://www.mdpi.com/article/10.3390/ijerph19158922/s1, file S1: Organizational steps; file S2: Original questionnaire before starting the distance seminar (pre), translated into English; file S3: Original questionnaire after finishing the distance seminar (post), translated into English.
