# Supplementary material for: Patient Care via Video Consultations: Piloting and S.W.O.T. Analysis of a Family Medicine Digitally Synchronous Seminar for Medical Students
Source: Int J Environ Res Public Health. 2022 Jul 22;19(15):8922. doi: 10.3390/ijerph19158922 (PMC9332513; doi:10.3390/ijerph19158922)
Supplement: Supplementary file 1 [file ijerph-19-08922-s001.zip › additional file S1 1_organisation steps.pdf]

The PDF file contains a more detailed outline of the organizational steps and templates for documents to replicate the seminar.

Organizational steps (generalized according to the pilot project)

Week (time calculation after seminar start)	organizational steps
- 21	Obtain agreement from the university and teaching practice
- 17	Expert meeting to consult didactic approach, set learning objectives, create student questionnaire
-11	Apply for funds, obtain quotes from video consulting companies
-9 till -7	Recruit student participants, ask for patients suggested by the doctor
-6 till -3	Educate patients and students, obtain consents, coordinate appointments, distribute information document (comment 1), collect questionnaire on student learning objectives and prior knowledge
	Purchase accounts for students and faculty, clarify details with video service providers
-2	Send dates
-2	Try out technology
-1	Upload files to the platform:<br>- pseudonymized patient files as PDF document (comment 2)<br>- Templates for the implementation of a DMP<br>- List of assignment of which students talk to which patients (pseudonymized)<br>- Link to the digital whiteboard<br>- Test audio of the digital stethoscope
0 till 4	Conduct seminar, update files, clarify students' organizational issues
5 till 13	Collect evaluation
5	Delete data (patient files, etc.)
5	Issue certificates of achievement, debriefing between instructors

Comment 1: Information to the students.

This document included:

- List of seminar dates
- Link to the questionnaire
- Declaration of consent
- Privacy Policy

- Declaration of confidentiality
- Structure of the group appointments and individual appointments
- Nomination of the participants
- Declaration for access to the group room of the video platform
- Link to the support of the video service provider
- Enumeration of practice equipment
- Request for preparation by means of files

Comment 2: Structure of a patient file.

The contents of the chapters can be inserted from screenshots from the patient's file from the practice management system. The document for the students can be created as a text file and saved as a password protected PDF document.
