# Supplementary material for: Patient Care via Video Consultations: Piloting and S.W.O.T. Analysis of a Family Medicine Digitally Synchronous Seminar for Medical Students
Source: Int J Environ Res Public Health. 2022 Jul 22;19(15):8922. doi: 10.3390/ijerph19158922 (PMC9332513; doi:10.3390/ijerph19158922)
Supplement: Supplementary file 1 [file ijerph-19-08922-s001.zip › additional file S2.pdf]

Page 01

DE13

Why have you decided to participate at the family medicine distance seminar?

Page 02

DE14

Please access your intrest

	strongly agree				strongly disagree
I am interested in family medicine	○	○	○	○	○
I am interested in video consultations	○	○	○	○	○

How do you estimate your prior knowledge in regard to the fellow students in your semester?

	strongly agree				strongly disagree
I know a lot about family medicine.	○	○	○	○	○
I know a lot about video consultations.	○	○	○	○	○

What do you aim to learn about family medicine during such seminar?

What do you aim to learn about video consultations during such seminar?

Is there anything else, you would like to learn during the seminar?

Within your study in medicine, how likely do you...	a lot				not at all
...participate at classes?	○	○	○	○	○
...want to be tought by working with real patients?	○	○	○	○	○
...want o participate from home (distance learning)?	○	○	○	○	○
...want to to participate in distance learning with real patients?	○	○	○	○	○
...want to be tough by a mixture of distance learning and classroom teaching?	○	○	○	○	○

Which groups of persons among medical students are interested in such distance learning from home with remote contact to patients?

Which semester do you attend?

[Please choose]

Do you have comments or suggestions for additions of the questionnaire?

We are thankful for every suggestion.

Last Page

Thank you very much for your participation!
