# Supplementary material for: Patient Care via Video Consultations: Piloting and S.W.O.T. Analysis of a Family Medicine Digitally Synchronous Seminar for Medical Students
Source: Int J Environ Res Public Health. 2022 Jul 22;19(15):8922. doi: 10.3390/ijerph19158922 (PMC9332513; doi:10.3390/ijerph19158922)
Supplement: Supplementary file 1 [file ijerph-19-08922-s001.zip › additional file S3.pdf]

Page 01

RU1

What did you learn within the seminar?

eg. about general practice, video consultations and working techniques

What did you NOT like within the distance seminar?

What was particularly good within the distance seminar?

Due to the patient interviews, my intrest in family medicine is...

- ○ ...decreased
- ○ ...remained constant
- ○ ...increased

Due to patient discussions, my intrest in remote treatments is...

- ○ ...decreased
- ○ ...remained constant
- ○ ...increased

The distance seminar...

...is an appealing method of distance teaching.

strongly disagree — strongly agree

...complements my previous teaching about family medicine.

strongly disagree — strongly agree

...complements my previous teaching on telemedicine.

strongly disagree — strongly agree

The connection to the consultation within the practice supported my learning process

strongly disagree	strongly agree

The conversation within the video consultation with patients in their home environment supported my learning process

strongly disagree	strongly agree

There was a good working atmosphere within the distance seminar.

strongly disagree	strongly agree

All in all, attending the event had been worthwile for me.

strongly disagree	strongly agree

How much working time did you invest in the preparation and follow-up of the patient consultation in which you were asked to lead the conversation?

[Please choose]

How much working time did you invest in the preparation and follow-up for every other appointment?

[Please choose]

Please evaluate the following statements about the distance seminar by comparising to other courses.

The workload for the students has been...

- too high
- just right
- too low

The level of performance for the students has been...

- too high
- just right
- too low

Do you have suggestions for improvement for the distance seminar?

Which components should be integrated into the distance seminar in the future?

The distance seminar should also be offered after the COVID-19 pandemic.

strongly disagree — strongly agree

What is the optimum number of participants for the distance seminar?

number

comments

What is the minimum semester for attending the distance seminar by medical students?

semester

What should future participants bring with them? (eg. previous knowledge, furnishings, attitude)

FA1

How much seminar meetings did you attend?

[Please choose]

How did you led your patient interview within the seminar?

[Please choose]

Do you want to make a proposal for supplements within this questionaire?

We are grateful for every suggestion.

Are there any aspects of the learning intervention, that has not been considered?

Last Page

Thank you very much for your participation!
